# State of Charge Dependent Mechanical Integrity Behavior of 18650 Lithium-ion Batteries

**DOI:** 10.1038/srep21829

**Published:** 2016-02-25

**Authors:** Jun Xu, Binghe Liu, Dayong Hu

**Affiliations:** 1 Department of Automotive Engineering, School of Transportation Science and Engineering, Beihang University, Beijing, China, 100191; 2 Advanced Vehicle Research Center (AVRC), Beihang University, Beijing, China, 100191; 3 Beijing Key Laboratory for High-efficient Power Transmission and System Control of New Energy Resource Vehicle, Beihang University, Beijing 100191, China; 4 Department of Aircraft Airworthiness Engineering, School of Transportation Science and Engineering, Beihang University, Beijing, China, 100191

## Abstract

Understanding the mechanism of mechanical deformation/stress-induced electrical failure of lithium–ion batteries (LIBs) is important in crash-safety design of power LIBs. The state of charge (SOC) of LIBs is a critical factor in their electrochemical performance; however, the influence of SOC with mechanical integrity of LIBs remains unclear. This study investigates the electrochemical failure behaviors of LIBs with various SOCs under both compression and bending loadings, underpinned by the short circuit phenomenon. Mechanical behaviors of the whole LIB body, which is regarded as an intact structure, were analyzed in terms of structure stiffness. Results showed that the mechanical behaviors of LIBs depend highly on SOC. Experimental verification on the cathode and anode sheet compression tests show that higher SOC with more lithium inserted in the anode leads to higher structure stiffness. In the bending tests, failure strain upon occurrence of short circuit has an inverse linear relationship with the SOC value. These results may shed light on the fundamental physical mechanism of mechanical integrity LIBs in relation to inherent electrochemical status.

Lithium–ion batteries (LIBs) have become the most popular commercial choice as power source for non-gasoline vehicles^1^,^2^,^3^. The crashworthiness design of electric vehicles (EVs) and hybrid EVs (HEVs) is widely pursued with fast growth in the EV and HEV market^4^,^5^. In particular, the mechanical integrity of LIBs serves as the governing indicator of crash safety^6^ has become an attractive problem in material science as well as electrochemical and mechanical engineering. In open literatures, pioneering work focusing on the mechanical integrity of LIBs has mainly studied the mechanical behavior of LIBs based on classical theories of mechanics^7^,^8^,^9^,^10^,^11^ and finite element analysis^11^,^12^,^13^,^14^,^15^,^16^,^17^. Efforts have been made to understand the mechanical behavior of LIBs subjected to various external mechanical loadings, such as radial compression^7^,^11^,^16^, indentation^11^,^12^, and bending^11^,^13^ on cylindrical^7^,^11^ and rectangular^7^,^15^,^16^ LIB shapes to derive the relationship between mechanical behavior and battery electrochemical effectiveness^7^,^11^,^14^,^15^. Some useful mechanical integrity criteria have been suggested to predict the occurrence of short circuit^11^.

LIBs undergo continuous charging and discharging cycles during operations, e.g., EVs running. Thus, understanding the mechanical behavior of LIBs at various values of state of charge (SOC) is extremely important because vehicle crashes usually occur when SOC varies during driving. Cannarella and Arnold^6^,^18^ found that the stack stress in battery cell is relevant to both SOC and state of health, and may be employed to monitor and evaluate the battery electrochemical status. The electrochemical status of the battery may influence the mechanical behavior of LIBs and the components to some extent.

Theoretical^19^,^20^,^21^,^22^ and experimental works^23^,^24^,^25^ on the mechanical behavior of electrochemically lithiated silicon with various lithium contents have been conducted to avoid the problematic fade shown by silicon anodes fracture processes during charging/discharging cycles. The results have proven that insertion of lithium–ion in anodes may cause elastic softening of the silicon anode, probably because of the formation of Li-rich areas in the grain boundary regions^26^. This finding further supports our idea to examine the electrochemically dependent mechanical behavior of LIBs and investigate the short circuit occurrence at various SOC values subjected to extreme mechanical loadings.

In the abovementioned references, evidences show that the mechanical properties of LIBs may change in various SOC values. The exact and quantitative relationships between mechanical behaviors and SOC status, as well as the SOC dependent mechanical integrity behaviors, are still lacking, which leads to shallow understanding of the crash safety of LIB. In this paper, the characteristic parameter of short-circuit is first determined and confirmed by examining the mechanical integrity of the 18650 LIB cell. Further, mechanical behaviors at various SOCs subjected to bending and compression are studied, and the quantitative mechanical integrity behaviors are obtained. Relationships of failure strain/stress to the SOC values at both compression and bending loadings are established.

## Results and Discussions

### Typical short circuit behaviors at compression loadings: At a fixed SOC value

**Figure 1:**
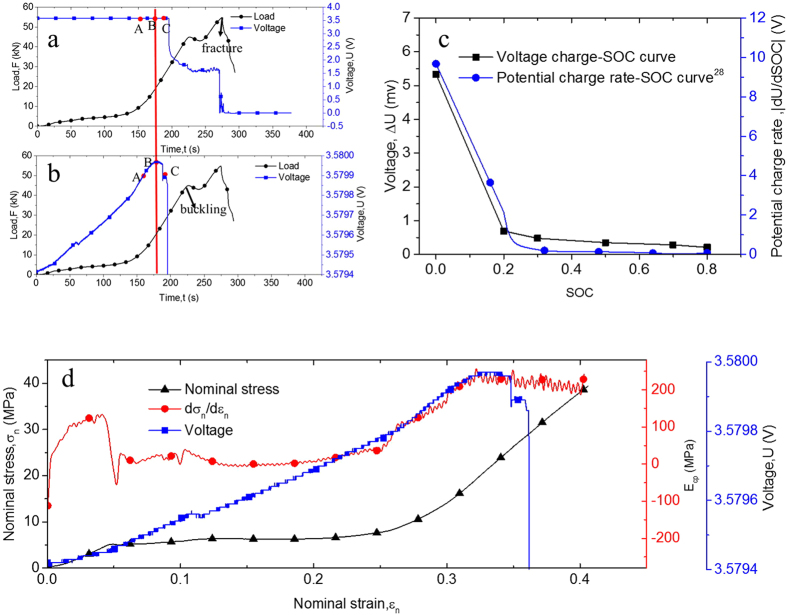
(**a**,**b**) Typical voltage-time and force-time curve in the compression test for 18650 LIB at SOC = 0.2. (**c**) Voltage change–SOC curve (maximum rising voltages in different SOC values during compression tests) and potential charge rate–SOC curve in Ref (28). (**d**) Illustration of the nominal stress–strain, derivative nominal stress-strain, and voltage-strain curves in compression test for 18650 LIB at SOC = 0.2.

The intact 18650 LIB cell was used for the quasi-static radial compression tests. Fig. 1(a) shows a typical curve for load-time and voltage-time history in radial compression at 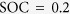 extracted from repeated experiments. The reaction force on the LIB would first increase gently because of the possible gaps between the battery skin and the jellyroll, within the innermost hollow separator rolling rod, as well as among the jellyroll layers. After densification, the force would increase drastically because the structure becomes stiff. The first force drop was caused by battery shell buckling, whereas the second force drop was mainly caused by skin fracture. A similar phenomenon was reported by Sahraei *et al*.^7^, which may serve as a support for the validity of our experiments. The mechanical behavior of different types of cells may use the same constitutive form with slightly different parameters because of the following reasons. First, the main material of the shell of the 18650 cells is steel or aluminum and the thicknesses of the shell vary because of design and manufacturing. Constitutive behaviors of aluminum and steel can both be expressed by Johnson-Cook model, which is expressed as 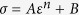 (where 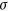 is the plastic stress, 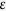 is the plastic strain, 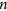 is the hardening exponent, and *A* and *B* is the parameters to fit); hence, they may share the same mechanical behavior form (which means the load-displacement curve shape is similar in the same loading conditions). Moreover, the shell is thin enough that the thickness change would not change the form. Second, the major components of the jellyroll are porous materials that also share the same mechanical behavior form, although the materials and the thicknesses of the anode, cathode, and separator may be different. In previous studies^11^,^12^,^14^, the mechanical model of the jellyroll is given as 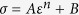 or 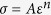, confirming that the parameters *A* and *B* by compression tests can describe the mechanical behavior of jellyrolls. The mechanical properties of the cell differ by capacities. Given the power and energy design requirement, the materials and dimensions may vary in 18650 LIBs, leading to different mechanical behaviors. Without knowing the exact material system and dimensions of all the cell components, the exact mechanical properties of the cell cannot be determined. Meanwhile, for example, if more active materials are added on the anode and the cathode to increase the capacity of the battery, higher modulus of the cell can be achieved.

For electrochemical behavior, initial short circuit may be directly reflected by the start of voltage drop^11^,^12^. To consolidate this judgment, three compression tests for LIBs with initial voltage of 3.44 V that stop at points B, A, and C (B is the point where the voltage starts to drop; A and C are the points before and after point B), as indicated in Fig. 1(a,b), are conducted. The axis shown in Fig. 1(b) was rescaled to assist the voltage variation profile. The tested samples were collected and set aside for 24 h. The voltages were measured as 3.44, 0, and 0 V for points A, B, and C respectively. Therefore, the drop of voltage should promptly indicate the short circuit of LIBs. The short circuit occurs at point A, before shell buckling is exhibited from a structure mechanics point of view. However, in the hemisphere punch test (which is similar to indentation), the initial short circuit occurred simultaneously when the reaction force reached its peak value^12^, quite different from that observed during the compression tests in Fig. 1(b). During compression, the voltage would increase slightly, probably because of the change in electrochemical properties of the cell caused by external force^27^. Although trivial, the voltage increase 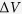 is dependent on SOC value, as illustrated in Fig. 1(c). 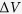 decreases with SOC values. Generally, voltage changes because of the electrode potential variation. In this paper, the active material of the cathode is 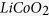, while the material of the anode is graphite. The electrode potential of these two materials have been studied previously^28^,^29^. The potential of the graphite decreases with the increase in SOC because of the insertion of Li^+^. Under compression condition, a small amount of Li^+^ may insert to the graphite, thereby slightly decreasing the potential. Assuming that the insertion number of Li^+^ is constant, the change potential of graphite will be larger in low SOC cells. Fig. 1(c) shows the potential change rate 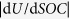 of graphite from Ref.^28^, and the trend of the curve is similar to the voltage change–SOC curve.

To further quantify the short circuit occurrence with mechanical behavior of the battery cell, the nominal stress–strain curve depicting mechanical behavior has been proposed. Nominal stress 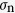 is considered as


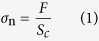


where 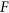 is the force, the same as that in Fig. 1(a); and 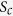 refers to the contact area calculated as follows:


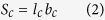


where 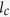 is the length of the cell, and the contact width *b*_*c*_ can be further obtained as follows:


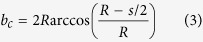


where *R* denotes the radius of the cell and *s* is the displacement of the indentor. Accordingly, the nominal strain 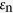 can be expressed as follows:


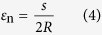


**Figure 2:**
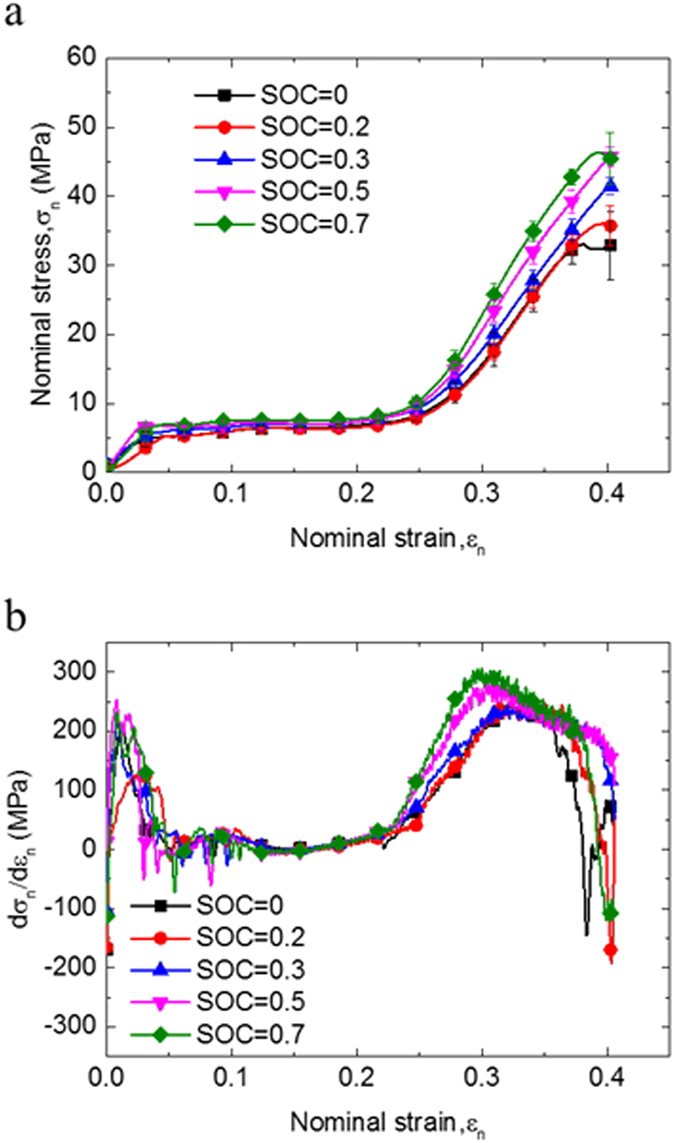
(**a**) Nominal stress–strain curves in various SOCs and (**b**) stress gradient curve in various SOCs during compression tests.

The nominal stress–strain curve is the black dot line shown in Fig. 1(d). The nominal stress–strain curve can be analyzed to that of the porous materials^30^; thus, point A can be regarded as the densification starting point. The initial short circuit can be associated with the densification of the cell. LIB equivalent compression modulus 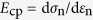 is expressed as the red curve in the same picture. The point with the maximum value of 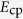 matched with point A where the short circuit occurs. After internal short circuit, the temperature of the cell increases from 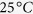 to 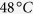 in low SOC within 30 s^12^ and almost 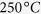 within 18 s^31^ in high SOC. 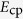 decreases gradually because of the temperature rise caused softening although the force and nominal stress both continued their growth caused by changing the mechanical properties of the cell components at different temperatures. The DMA tests in Ref. 32 showed that the modulus of the separator decreased within the temperature range of 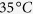 to 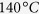. In our recent study^33^, the modulus decrease temperature range was 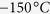 to 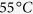. Furthermore, mechanical studies^34^,^35^,^36^ showed that the shell material (steel) and the collector material (copper and aluminum) also became softer at high temperature. Given that temperature will rise faster in high SOC cells after initial short, faster softening in high SOC cells can be observed in Fig. 2(b). Thus, 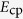 can be an important and direct index for the real-time monitoring of short circuit onset.

### Short circuit behaviors at compression loadings: At various SOC values

**Figure 3:**
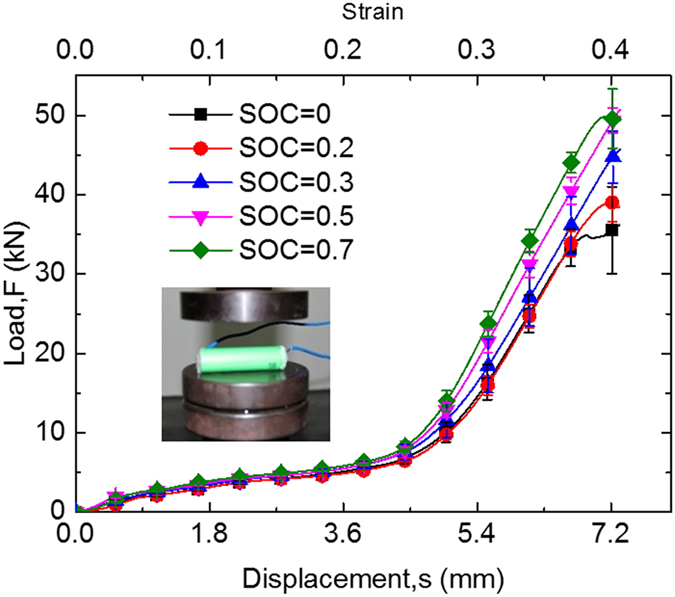
Different mechanical behaviors of 18650 LIB at SOC value of 0 to 0.7 during compression tests.

LIB cells with SOC values from 0 to 0.7 were tested at different compression loadings. Force-displacement curves with similar profiles may be extracted from Fig. 1(a) to be shown in Fig. 3. The repeated tests were in good consistency and confirmed that cells become stiffer with higher SOC value. These results are qualitatively supported by the findings in Ref.^12^. No quantitative comparisons have been conducted because different types of 18650 cell have been chosen for the study. The hardening of the cell structure occurred because of internal electrochemical reaction. In nanoscale, charging leads to insertion of Li^+^ in the anode, while deintercalation in the cathode. However, no literature with nanoindentation experiment results for both graphite (cathode material) and LiCoO_2_ (anode material), with or without lithium ions, is available.

**Figure 4:**
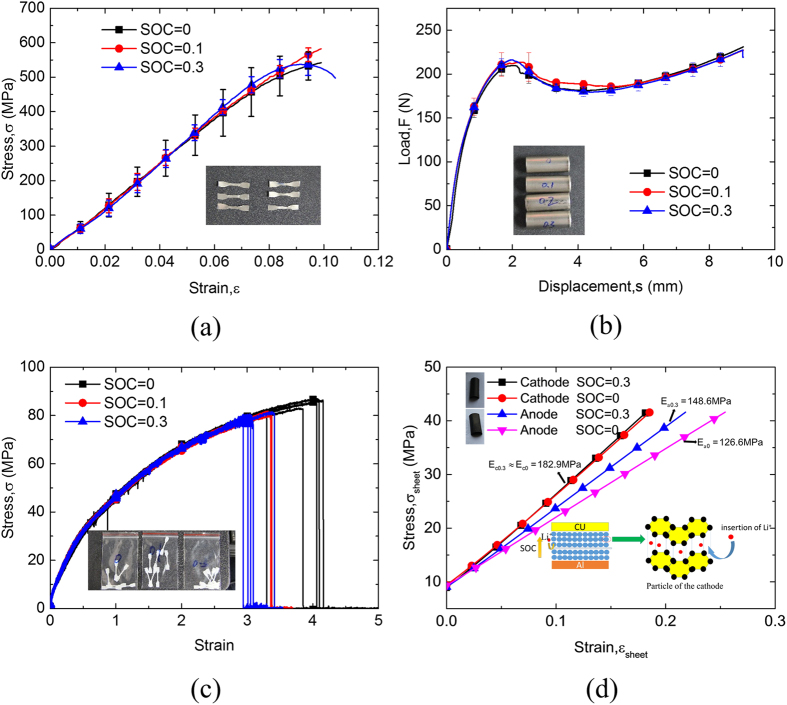
(**a**) Stress–strain curves for the tension tests of the skin at different SOCs. (**b**) Load-displacement curves for the compression tests of the skin at different SOCs. (**c**) Stress–strain curves for the tension tests of the separator at different SOCs. (**d**) Stress–strain curves for the cathode and anode during compression in two different SOCs with schematic for the intercalation of Li^+^. Note that *E* presents the bending modulus. Subscripts “a” and “c” refer to anode and cathode, respectively. Numbers in the subscripts indicate the SOC values.

The major mechanical components of LIBs include LIB shell, cathodes, and anodes with active material and separator. The abovementioned key mechanical components of the cell were tested for the mechanical properties in various SOC values to illustrate the hardening of LIBs at higher SOC value. First, the shells of the LIB cells from SOC = 0 to SOC = 0.3 were cut into dog-bone-shaped specimen for the tension tests. The intact LIB shells with the same SOC values as those in the material testing were also compressed radially. No obvious SOC dependency can be observed in the results shown in Fig. 4(a,b). Similar results were obtained for the tension tests of LIB separators from various SOC values shown in Fig. 4(c). Thus, the SOC dependency brought by the shell and the separator can be ruled out. Finally, two types of cells in different SOCs (0 and 0.3) were disassembled. The cathode and anode layer sheets (with active material) were cut into rectangular shape having 54 mm length and 20 mm width. A total of 21 layers were stacked together, where the convergence study indicated that such thickness can rule out the effect caused by the geometric shape of the structure, reflecting the true mechanical behavior. First, the thickness changes with the change in SOC. The cathode layer does not change with the SOC value; however, the anode layer expands by about 9.8% from SOC 0 to SOC 0.3. The cathode layer is insensitive to the SOC value; however, SOC has a much stronger influence for anode layer, i.e., the compressive Young’s modulus 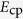 increases by almost 20% from the cases with SOC = 0 to SOC = 0.3, as shown in Figs. 4(d). From a nanoscale point of view, insertion of Li^+^ makes the graphite structure stiffer, where additional bond may form between Li^+^ and carbon atoms. Ref. [37] reported that the insertion phenomenon of Li+ causes the initial stress of the anode particle, which can be the supportive reason to make the particle stiffer. Ref. [38] used the density functional theory (DFT) to reveal that the Young’s modulus of graphite tripled as it is lithiated to LiC_6_, and the Young’s modulus have a liner relationship with the lithium concentration.

**Figure 5:**
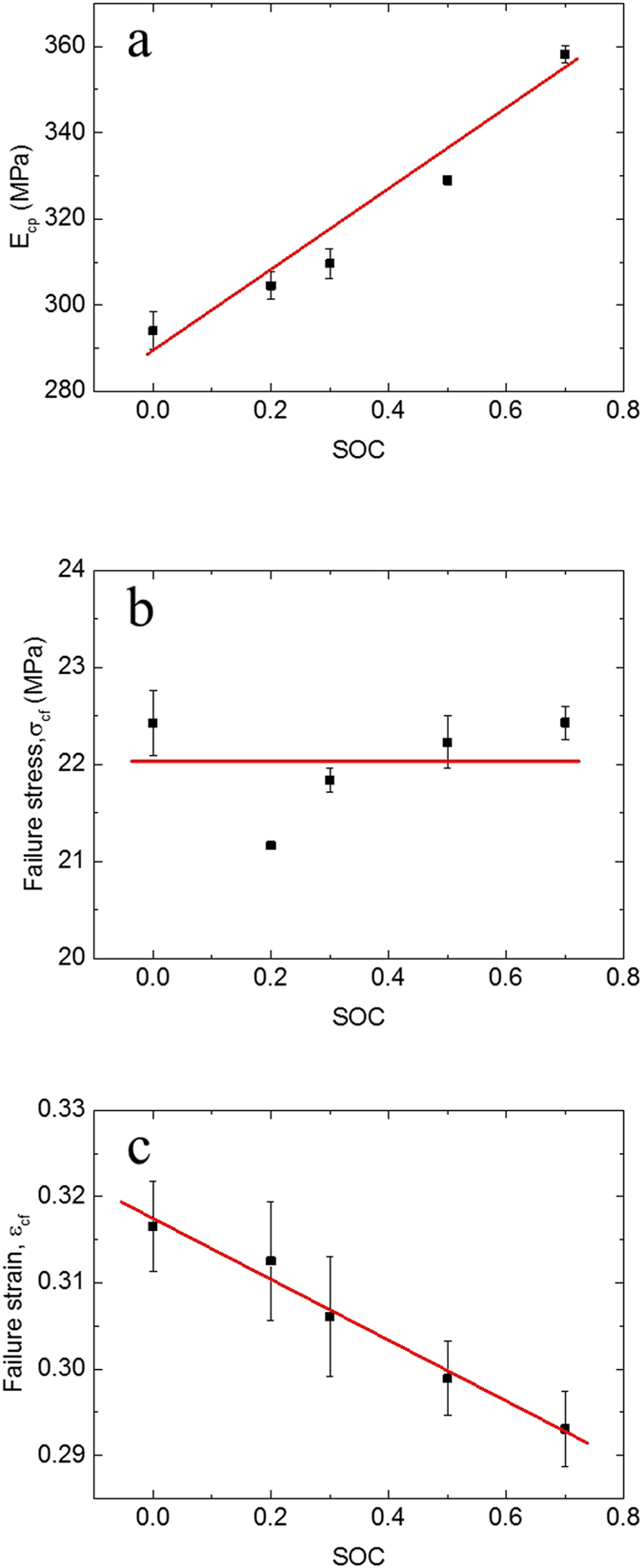
Relationship between SOC and (**a**) maximum structural stiffness 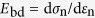 (**b**) Nominal failure stress 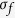 and (**c**) Nominal failure strain 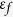.

In the above sections, a strong correlation was observed between the maximum of the equivalent compression modulus 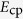 and the onset of the initial short circuit. Thus, the mechanical integrity failure strain 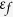 (nominal strain when the short circuit occurs) at various SOCs can be summarize. The experimental data in Fig. 3 were extracted and converted to the nominal stress–strain curve according to Eqs. (1) to (4), 16, 18, 20, as shown in Fig. 2. Summary of the curves can be expressed in Fig. 5, where battery cells with high SOC failed at smaller 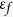, but larger maximum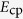. 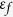 changes because of electrode expanding and propriety changes of the separator. On the contrary, the corresponding nominal failure stresses 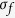 remain almost the same.

These approximately linear relationships can be fitted and expressed as follows:


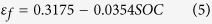



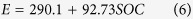



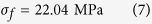


### Short circuit behaviors under bending tests: At various SOC values

**Figure 6:**
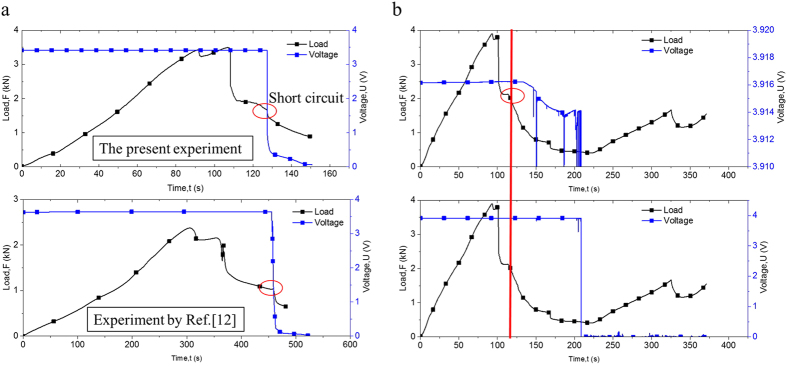
(**a**) Load and voltage-time curves for 18650 LIB under three-point bending tests with SOC = 0.1 and compared with Ref. [12]. (**b**) Load and voltage-time curve for 18650 LIB under three-point bending tests with SOC = 0.7.

LIB cells with SOC values from 0 to 1 were tested in three-point bending tests. The mechanical behavior of the LIB cell is quite different from that under compression. The compression tests mainly reflect the mechanical properties in the radial direction, whereas the bending tests represent more complicated properties of the cell, where the upper layer sustains compression while the lower layer experiences tension. The reaction force increases almost linearly in the beginning, and the equivalent bending modulus may be obtained by 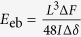 (Note that 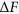 and 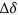 is the changed reaction force and displacement from experiment results, respectively), where 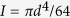 (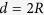 is the diameter of the cell) and obtain 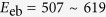 MPa under various SOCs. After the force reaches its peak value, battery shell buckling occurs, which will be followed by battery shell fractures. Qualitatively similar force and voltage time history profiles were obtained in Ref. [12] for low SOC values. However, the voltage behaviors were quite different: for low SOC value cases, the voltage would drop drastically to zero when short circuit occurs, whereas, in high SOC value cases, the voltage would drop gradually and continuously. After 90 s in the current experiment shown in Fig. 6(b), the voltage suddenly dropped to zero. Thus, higher SOC may help to sustain the voltage even after short circuit occurrence for quite some time, although the short circuit current is the same for the cells with high and low SOC. The low SOC cell will see a faster drop in voltage because the electrons will be depleted faster from the electrodes; whereas, the high SOC cell will have a much slower drop in voltage because it takes time for the short circuit to complete. Notably, fast voltage drops caused by the sudden shock of the sample were observed during testing when the fracture propagates.

**Figure 7:**
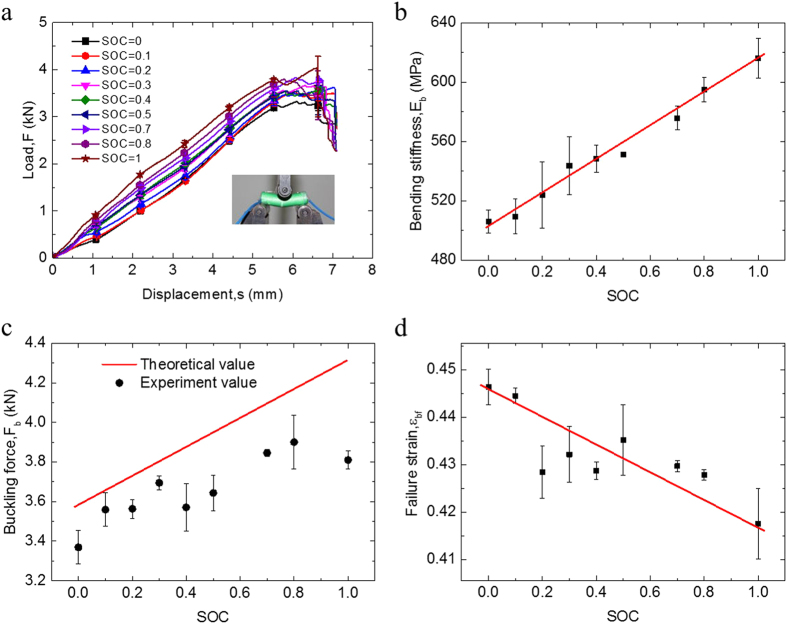
(**a**) Load-displacement curves of 18650 LIB cells with SOC values from 0 to 1 under bending. (**b**) Relationship between SOC and bending modulus. (**c**) Relationship between SOC and buckling force. (**d**) Relationship between SOC and failure strain 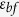.

From the systematic SOC-dependent mechanical behavior of LIB under bending tests shown in Fig. 7(a), the bending stiffness of the cell can be associated with SOC. Similarly to the compression test results, cells with higher SOC have higher bending stiffness *E*_b_. A linear relationship can be fitted and established as follows:


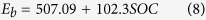


The first drop of the load is caused by the buckling of the shell. The following equations^39^,^40^,^41^ can be used to predict the ultimate bending capacity 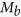 of the shell, the corresponding angle of rotation 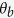, and the loading displacement 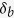, which is the displacement when buckling occurs. Written as


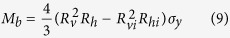



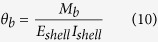



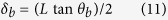


where 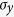 is the measured yield stress of the shell equal to 320 MPa [Fig. 4(a)], 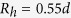, 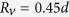, 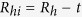 (*t* is *t*he thickness of the shell, which is 0.3 mm), 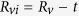, 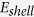 is the modulus of the shell, which is 207 GPa, and 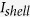 is the inertia moment of the shell equal to 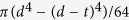. The buckling force can then be calculated by 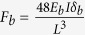 (*E*_*b*_ is established by Eq. (8) and 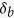 is confirmed by Eq. (9, 10, 11)), which is given by


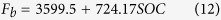


Fig. 7(b,c) show the bending stiffness *E*_*b*_and buckling force F_*b*_ in various SOCs. The theoretical value of the buckling force is slightly larger than the experiment value, indicating that the established bending stiffness is larger than the actual value.

In the bending case, the failure strain 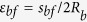 (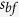
is the displacement when initial short happens and *R*_*b*_ = *R* is the radius of the cell) is also linearly dependent on SOC, as shown in Fig. 7(d), with the relationship expressed as follows:


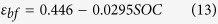


However, the discrepancy of the data points away from the linear relationship and from the engineering point of view, which is still acceptable by examining the data distribution. The actual difference of 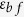 between the maximum and minimum SOC values, i.e., SOC = 0 and SOC = 1 is less than 10%, such that a simple linear function may well fit and reflect the relationships.

## Concluding Remarks

SOC-dependent mechanical integrity behavior is important in studying the crashworthiness of LIBs, especially at the working situation when charging and discharging occur, leading to the change in SOC value. In this study, the SOC-dependent mechanical integrity behavior was investigated at both compression and three-point bending tests. In the compression tests, LIB cells exhibited mechanical hardening mechanism with the increase in SOC values, which was further explained by the anode and cathode layer compression tests. The value of nominal maximum compression modulus also agrees with the voltage drop point, and thus this modulus can be used as indicator for real-time short circuit detection. Nominal failure strain decrease linearly with the increase in SOC values, whereas nominal failure stress was SOC independent. Similarly, in the bending tests, the bending modulus increased with SOC, whereas the failure strain linearly decreased with SOC values. This research may serve as the first step in understanding the mechanical integrity behavior at working condition and also provides basic experimental results to guide crash-safety single battery and battery pack design and monitoring.

## Experiment methods

### Testing methods

**Figure 8:**
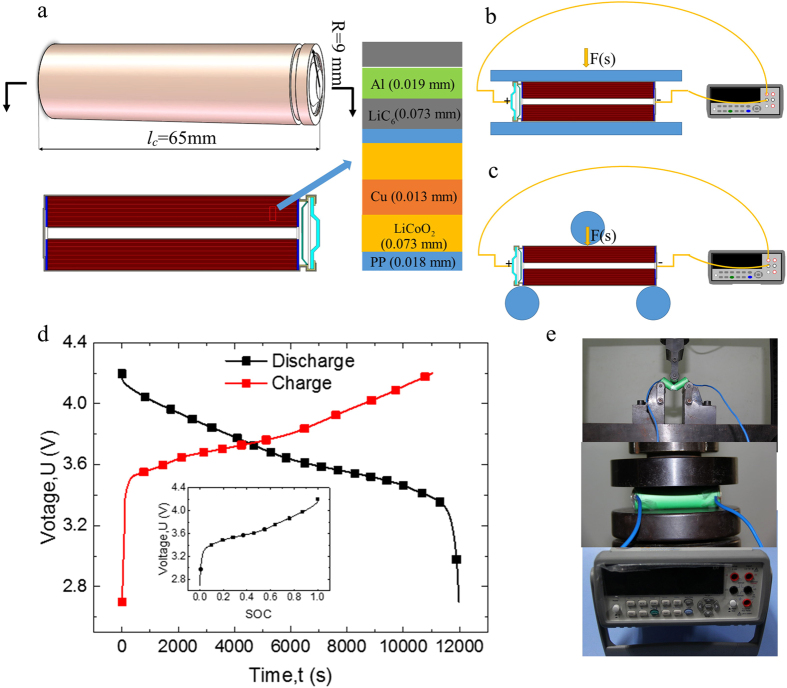
(**a**) Schematic of 18650 lithium–ion battery cell with geometry dimensions. (**b**) Compression test setups for 18650 LIB and (**c**) Three-point bending test setups for 18650 LIB. (**d**) Changing/discharging curve in 0.3 C and the voltage–SOC relationship. (**e**) Schematic of the experiment setups.

Given the quick short circuit of LIBs under axial compression and the difficulty in making a dog-bone shaped tension sample for LIBs, two typical mechanical loadings, i.e., compression and three-point bending tests, were selected to represent the possible external mechanical loadings imposed on LIBs. In the bending tests, the structures and materials undergo more complicated deformation, where the upper layer sustains compression and the lower layer experiences tension. INSTRON 5966 universal material testing machine was chosen to provide satisfactory mechanical testing platform for the tests with maximum loads of 100 kN and enhanced resolution of 50 N. The loading speed was set at 5 mm/min to provide quasi-static mechanical loadings, where strain rate and inertia effects can be ruled out. The voltage of LIB was measured *in-situ* by Agilent 34410A digital voltmeter, and the data were recorded at the frequency of 10,000 Hz, with an accuracy of 0.01 mV. Thus, voltage-time and force-displacement curves can be extracted. The temperature was not measured by this study because voltage was faster and more accurate method to detect the initial short. A safety shield made of polymethyl methacrylate was placed within the boundary of the testing machine to provide necessary protection against possible fire and explosion. Three repeated tests were conducted at each loading scenario to confirm the validity of the experimental results. Test samples were selected from SOC = 0 where no further discharging could be conducted to SOC = 1 where LIB is fully charged. For safety concerns, different maximum SOC values were used in different loading conditions, where no fire or explosion during experiments was ensured, e.g., the maximum SOC values were set as 0.8 and 1 for compression and bending, respectively. Compression and bending test setups are shown in Fig. 8(b,c).

### Testing samples

Without loss of generality, the most widely commercialized 18650 LIBs were chosen in this study. These cells were provided by the Japanese company SONY. The nominal capacity of the LIBs is 2250 mAh, nominal voltage is 3.7 V, change voltage is 4.2 V, and weight is 43.6 g. The schematic of the 18650 LIBs is shown in Fig. 8(a). This 18650 cell mainly consisted of shell and jellyroll, and the cylindrical jellyroll is wound with two layers of the separator, i.e., one layer of positive electrode and one layer of negative electrode. The shell case is made of steel with a thickness of 0.26–0.3 mm; the positive electrode material is 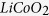 adhering to the aluminum collector. The negative electrode material is graphite glued to the copper collector, and the separator is made of polypropylene (PP). The thicknesses of these components are shown in Fig. 8(a). The charging/discharging curves and the voltage–SOC curve are illustrated in Fig. 8(d). The charge and discharge curves changed quickly before voltage 3.2 V or after voltage 4.1 V, and became stable during 3.2–4.1 V, as determined by the electrochemistry properties of the anode material graphite^28^ and cathode material 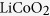
29.

## Additional Information

**How to cite this article**: Xu, J. *et al*. State of Charge Dependent Mechanical Integrity Behavior of 18650 Lithium-ion Batteries. *Sci. Rep*. **6**, 21829; doi: 10.1038/srep21829 (2016).
